# X-ray tomography data of compression tested unidirectional fibre composites with different off-axis angles

**DOI:** 10.1016/j.dib.2019.104263

**Published:** 2019-07-15

**Authors:** D. Wilhelmsson, L.P. Mikkelsen, S. Fæster, L.E. Asp

**Affiliations:** a Industrial and Materials Science, Chalmers University of Technology, SE-41296, Göteborg, Sweden; b Composite Mechanics and Structures, DTU Wind Energy, Technical University of Denmark, DK-4000 Roskilde, Denmark

## Abstract

This data article contains lab-based micro-computed tomography (μCT) data of unidirectional (UD) non-crimp fabric (NCF) carbon fibre reinforced composite specimens that have been deformed by compression. The specimens contain UD fibres with off-axis angles of 0°, 5°, 10°, 15° and 20° and the compression testing induces kink-band formation. This data formed the basis for the analysis of the influence of in-plane shear on kink-plane orientation as reported in Wilhelmsson et al. (Wilhelmsson et al., 2019).

**Table:** Specifications table

Subject area	*Materials Science*
More specific subject area	*Fibre composites, unidirectional, off-axis angle, X-ray tomography, compression damage mechanics, kink band*
Type of data	*X-ray tomography data*
How data was acquired	*Laboratory X-ray tomography scanner (Zeiss Xradia 520 Versa)*
Data format	*Raw and reconstructed X-ray CT*
Experimental factors	*Specimens contains UD fibres with off-axis angle of 0°, 5°, 10°, 15° and 20° that have been compression testing to kink-band formation*
Experimental features	*Five specimens have been tomographic scanned with a field of views* 3 mm *and* 13 mm *with a pixel size of 12.*77 μm *and 3.*02 μm*, respectively*
Data source location	*Roskilde, Denmark, Latitude: 55.695343, Longitude: 12.08921*
Data accessibility	*The data is available online at:* https://doi.org/10.5281/zenodo.1439209
Related research article	*The datasets presented in this paper have been used in**[1]**to determine the resulting kink-plane angles in an off-axis loaded unidirectional non-crimp fabric carbon reinforced composite. A novel three-dimensional finite element model was developed based on the true fibre misalignment angles obtained from the tomography datasets.* *[1]*D. Wilhelmsson*,*L.P. Mikkelsen*,*S. Fæster*and*L.E. Asp*. Influence of in-plane shear on kink-plane orientation in a unidirectional fibre composite. Composites Part A: Applied Science and Manufacturing,* 119 *(2019) 283–290.* https://doi.org/10.1016/j.compositesa.2019.01.018*.*

**Table:** 

**Value of the data** • The datasets contain detailed information about the kink-band formation and its dependency on the off-axis angles in a unidirectional fibre composite, and can be used to further the understanding of the damage mechanisms during compression. • The data represents the final morphology of the kink bands developed in a uniform gauge section. The data can serve as a baseline case for modelling of kink-band formation. • The data contain both a large and a small field-of-view allowing observation of parameters like fibre waviness both in-plane and out-of-plane. • The data-set can be used for developing segmentation algorithm for determination of kink-band failures in composite materials, the controlling failure mechanism during compression of uni-directional composite materials. • The dataset can be used for validation of the observations and conclusions reported in reference [1].

## 1 Data

**Fig. 1:**
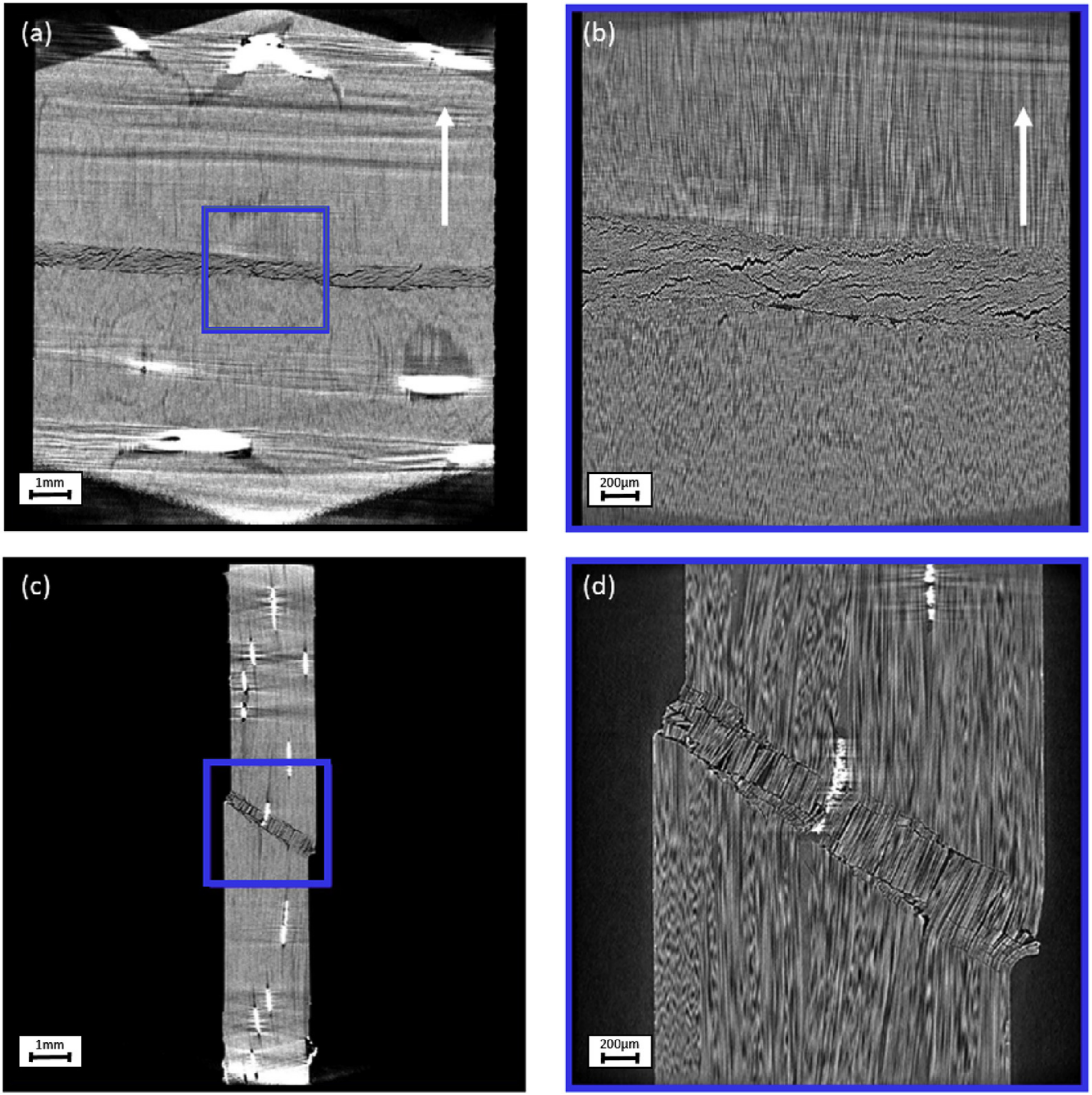
Cross sections through the reconstructed specimen with UD fibres with off-axis angles of 0° through (a) XY plane in FOV 13mm, (b) XY plane in FOV 3 mm, (c) XZ plane in FOV 13 mm, (d) XZ plane in FOV 3 mm. The blue box in (a) and (c) mark the position of the FOV 3 mm scan in (b) and (d). The fibre direction, indicating the off-axis angle, is marked in (a) and (b) with a white arrow.

**Fig. 2:**
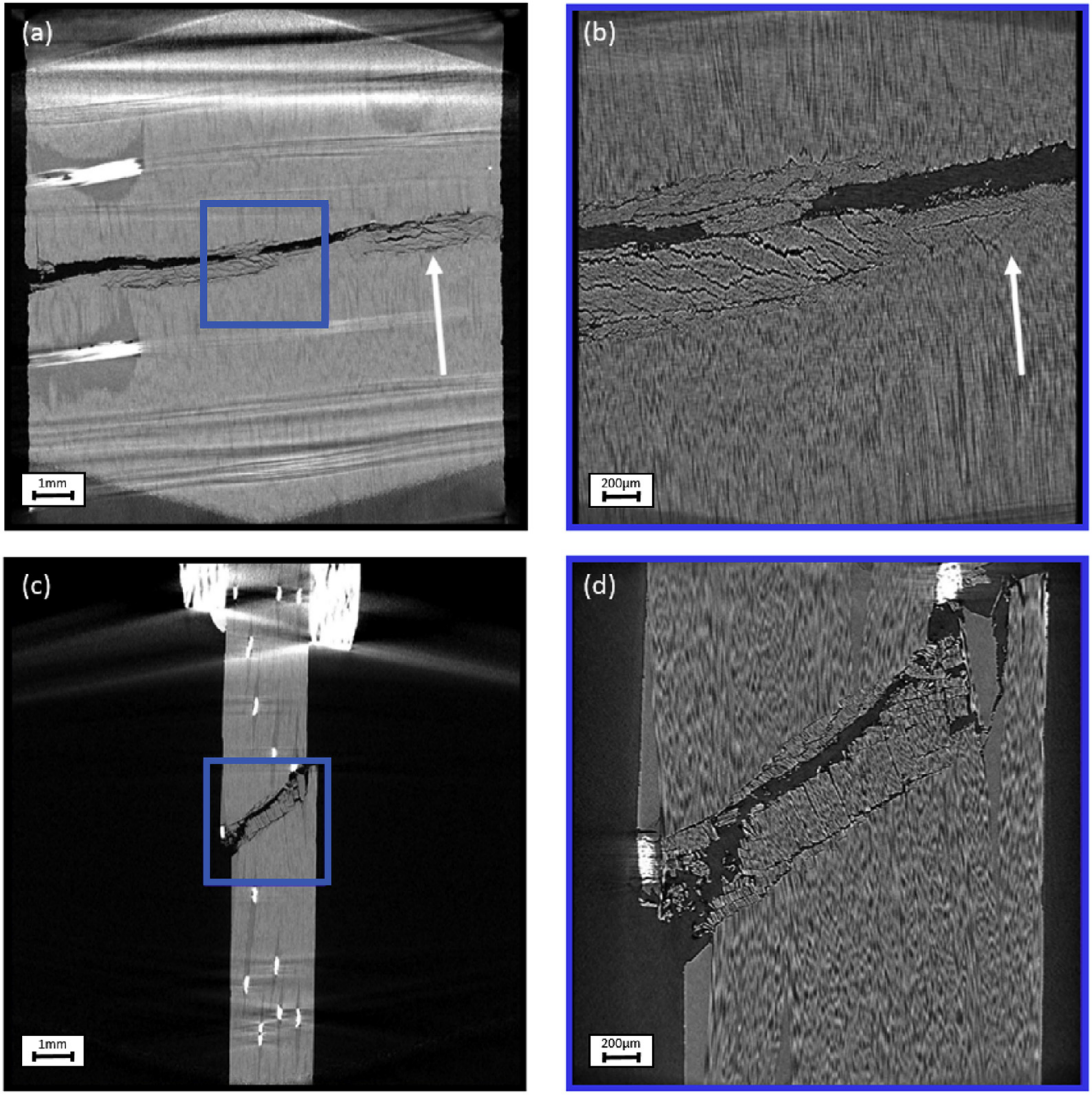
Cross sections through the reconstructed specimen with UD fibres with off-axis angles of 5° through (a) XY plane in FOV 13mm, (b) XY plane in FOV 3 mm, (c) XZ plane in FOV 13 mm, (d) XZ plane in FOV 3 mm. The blue box in (a) and (c) mark the position of the FOV 3 mm scan in (b) and (d). The fibre direction, indicating the off-axis angle, is marked in (a) and (b) with a white arrow.

**Fig. 3:**
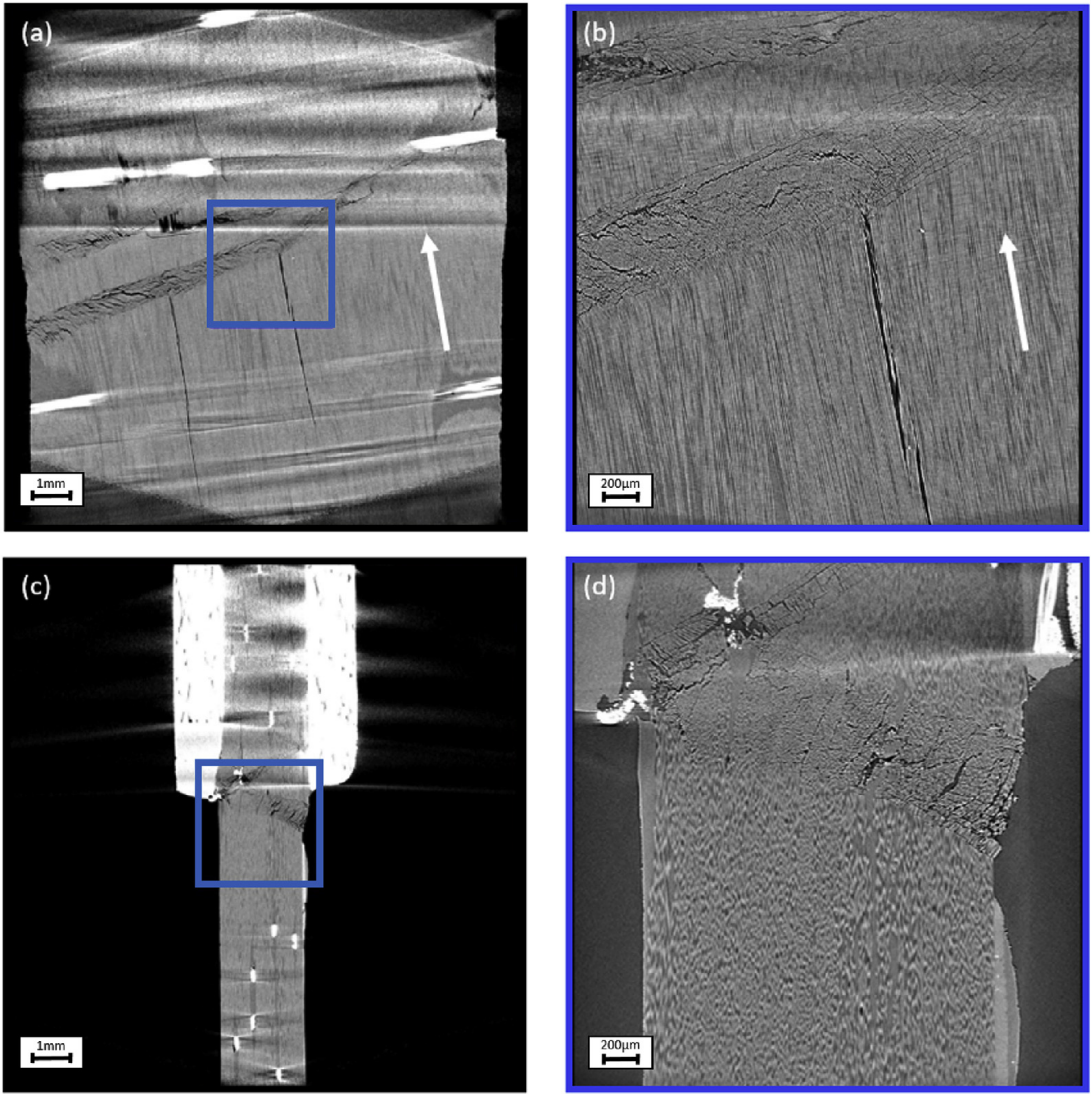
Cross sections through the reconstructed specimen with UD fibres with off-axis angles of 10° through (a) XY plane in FOV 13mm, (b) XY plane in FOV 3 mm, (c) XZ plane in FOV 13 mm, (d) XZ plane in FOV 3 mm. The blue box in (a) and (c) mark the position of the FOV 3 mm scan in (b) and (d). The fibre direction, indicating the off-axis angle, is marked in (a) and (b) with a white arrow.

**Fig. 4:**
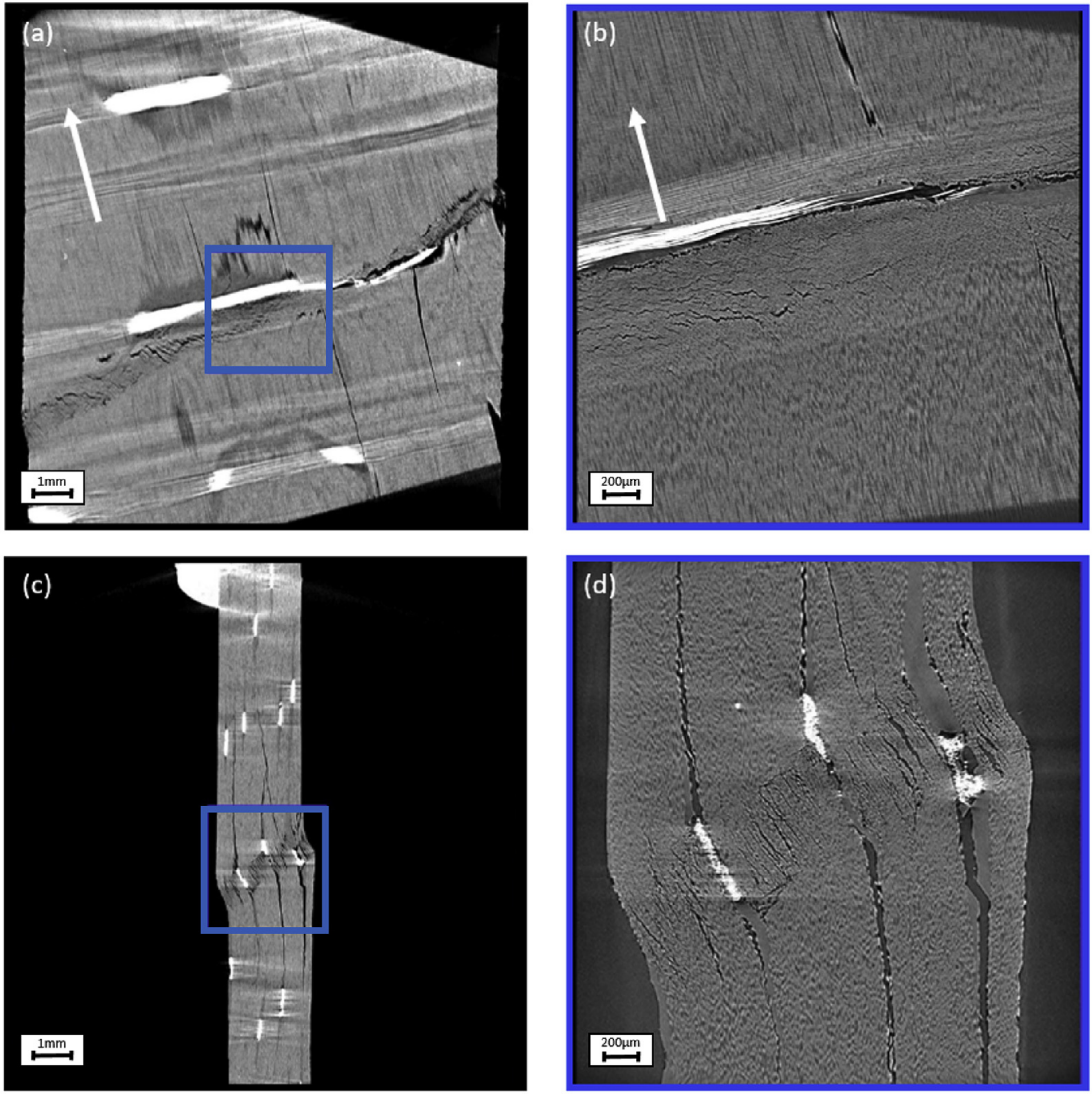
Cross sections through the reconstructed specimen with UD fibres with off-axis angles of 15° through (a) XY plane in FOV 13mm, (b) XY plane in FOV 3 mm, (c) XZ plane in FOV 13 mm, (d) XZ plane in FOV 3 mm. The blue box in (a) and (c) mark the position of the FOV 3 mm scan in (b) and (d). The fibre direction, indicating the off-axis angle, is marked in (a) and (b) with a white arrow.

**Fig. 5:**
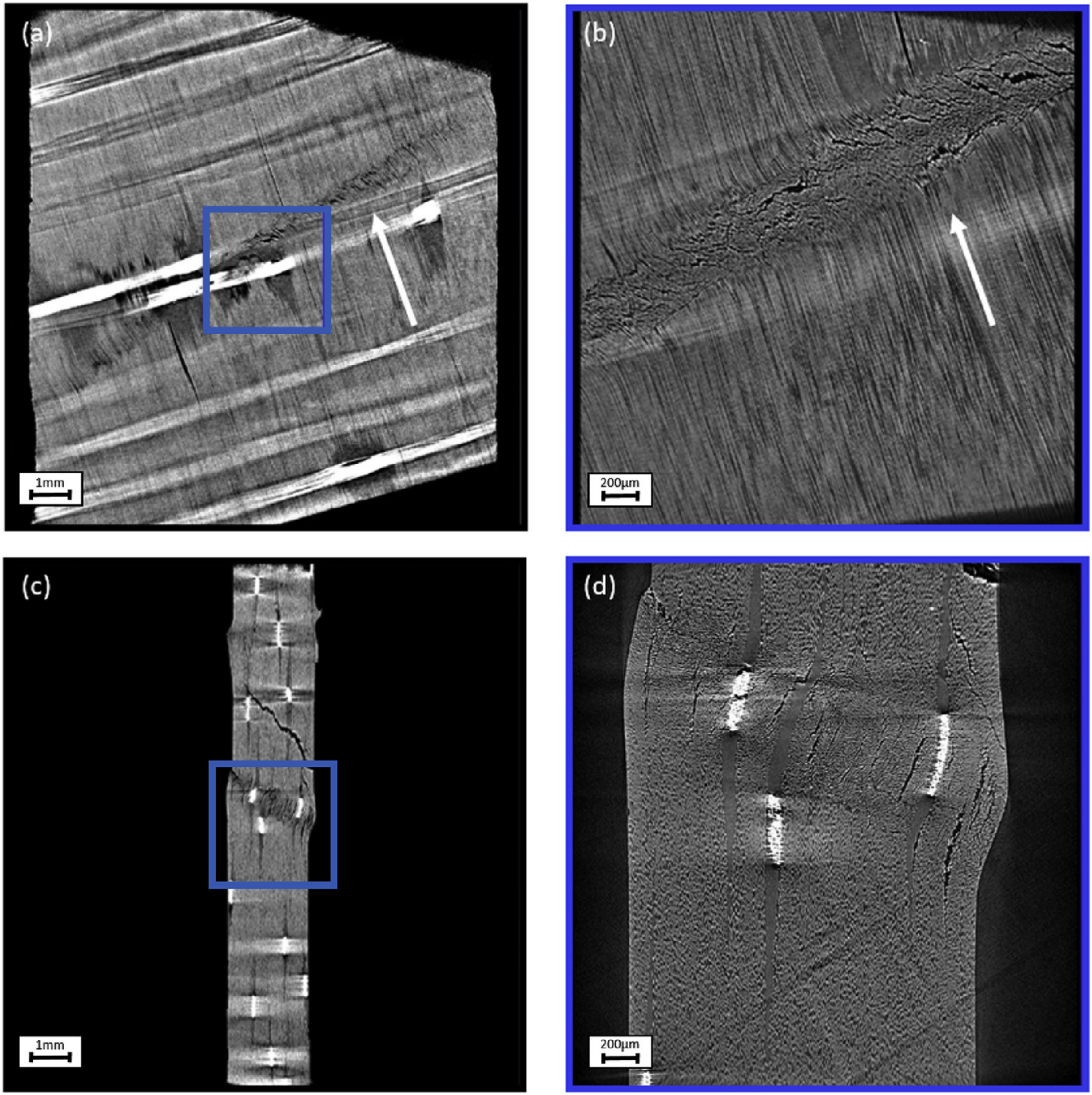
Cross sections through the reconstructed specimen with UD fibres with off-axis angles of 20° through (a) XY plane in FOV 13mm, (b) XY plane in FOV 3 mm, (c) XZ plane in FOV 13 mm, (d) XZ plane in FOV 3 mm. The blue box in (a) and (c) mark the position of the FOV 3 mm scan in (b) and (d). The fibre direction, indicating the off-axis angle, is marked in (a) and (b) with a white arrow.

The data presented in this paper consist of 10 X-ray tomography datasets of unidirectional (UD) non-crimp fabric (NCF) carbon fibre reinforced composites. The difference between the datasets is the off-axis angle of the UD fibres which are 0°, 5°, 10°, 15° and 20°. The off-axis angle is the angle between the UD fibres and the compression axis. The off-axis orientation is illustrated in the 5 cross sections through the reconstructions in Fig. 1, Fig. 2, Fig. 3, Fig. 4, Fig. 5. Each sample has been scanned with a field of view of 13 mm (FOV 13 mm) and a field of view of 3 mm (FOV 3mm). The raw projection data is in the “.txrm” format and the reconstructed data is available in both the “.txm” and the “.tif” format. The “.txrm” and “.txm” format are the regular output formats for the raw and reconstructed image data of the Zeiss Xradia 520 Versa system used for the data acquisition. The dataset also includes three movies of each reconstructed dataset in which the volumetric data is sectioned in the XY, XZ, YZ planes.

### 2 Experimental design, materials, and methods

The specimens that have been tomographically scanned consist of UD fibers with off-axis angles of 0°, 5°, 10°, 15° and 20°. The reader is referred to [1] for a detailed description of how the samples were manufactured. The tomography scans were performed on a Zeiss Xradia 520 Versa. The X-ray scanner was equipped with a tungsten target. An acceleration voltage of 30kV and a power of 7mA was applied to generate X-rays with energies up to 30 keV. Projections were acquired during a full 360° rotation of the specimens. The detector size was 2k × 2k and projection images with a binning of 2 were aquired to increase the signal to noise ratio. A Feldkamp reconstruction algorithm [2] for cone beam reconstructions were applied resulting in 3D reconstructions with voxel sizes of 12.77 μm and 3.02 μm for the FOV 13mm and FOV 3mm scans, respectively. All relevant scan parameters are listed in Table 1.

**Table 1:** X-ray tomography settings.

Parameter	FOV 13mm	FOV 3mm
Optical magnification	0.4X	4X
Source to sample distance (mm)	23	13
Detector to sample distance (mm)	100	16
Exposure time (sec)	12	10
No. of projections	5201	5801
Rotation	360°	360°
Accelerating voltage (kV)	30	30
Binning	2	2
Pixel size (μm)	12.77	3.02
Source filter	air	air
Reconstruction filter	0.5 smooth	0.5 smooth
Beam hardening correction	0.05	0.05
